# Docosahexaenoic Acid and Adult Memory: A Systematic Review and Meta-Analysis

**DOI:** 10.1371/journal.pone.0120391

**Published:** 2015-03-18

**Authors:** Karin Yurko-Mauro, Dominik D. Alexander, Mary E. Van Elswyk

**Affiliations:** 1 Clinical Research, Nutritional Lipids, DSM Nutritional Products, Columbia, Maryland, United States of America; 2 Epidemiology, EpidStat Institute, Evergreen, Colorado, United States of America; 3 Scientific Affairs, Van Elswyk Consulting, Inc., Longmont, Colorado, United States of America; Texas Tech University Health Science Centers, UNITED STATES

## Abstract

**Introduction:**

Subjective memory complaints are common with aging. Docosahexaenoic acid (DHA; 22:6 n-3) is a long-chain polyunsaturated fatty acid (LCPUFA) and an integral part of neural membrane phospholipids that impacts brain structure and function. Past research demonstrates a positive association between DHA plasma status/dietary intake and cognitive function.

**Objectives:**

The current meta-analysis was designed to determine the effect of DHA intake, alone or combined with eicosapentaenoic acid (EPA; 20:5 n-3), on specific memory domains: episodic, working, and semantic in healthy adults aged 18 years and older. A secondary objective was to systematically review/summarize the related observational epidemiologic literature.

**Methods:**

A systematic literature search of clinical trials and observational studies that examined the relationship between n-3 LCPUFA on memory outcomes in healthy adults was conducted in Ovid MEDLINE and EMBASE databases. Studies of subjects free of neurologic disease at baseline, with or without mild memory complaints (MMC), were included. Random effects meta-analyses were conducted to generate weighted group mean differences, standardized weighted group mean differences (Hedge’s g), z-scores, and p-values for heterogeneity comparing DHA/EPA to a placebo. *A priori *sub-group analyses were conducted to evaluate the effect of age at enrollment, dose level, and memory type tested.

**Results:**

Episodic memory outcomes of adults with MMC were significantly (P<.004) improved with DHA/EPA supplementation. Regardless of cognitive status at baseline, > 1 g/day DHA/EPA improved episodic memory (P<.04). Semantic and working memory changes from baseline were significant with DHA but no between group differences were detected. Observational studies support a beneficial association between intake/blood levels of DHA/EPA and memory function in older adults.

**Conclusion:**

DHA, alone or combined with EPA, contributes to improved memory function in older adults with mild memory complaints.

## Introduction

Docosahexaenoic acid (DHA; 22:6 n-3), is a long-chain omega-3 fatty acid (n-3 LCPUFA) and is an integral part of neural membrane phospholipids and as such impacts brain structure and function. Early in life DHA is rapidly accumulated by neural tissue contributing to development of the brain and eyes [1]. At the opposite of end of the age spectrum many, but not all, observational studies demonstrate a beneficial association between DHA and /or EPA blood levels/dietary intake and various aspects of cognitive function in older adults [2,3,4]. Results from clinical trials evaluating the effect of DHA on cognitive function are difficult to summarize however, due, at least in part, to the myriad of cognitive tests employed between studies and the diversity of subjects with regard to age and cognitive status. To date, meta-analyses of clinical trials have assessed composite memory scores or the results of individual memory tests, e.g. immediate recall, as opposed to a particular memory domain, and have considered data almost exclusively from trials of older adults [5,6,7,8]. A recent meta-analysis of composite memory scores reported in clinical trials supplementing DHA, alone or in combination with EPA (DHA/EPA), to older adults found no benefit on composite memory, but reported a significant improvement in immediate recall among individuals with mild memory complaints (MMC) [8]. Immediate recall specifically tests episodic memory [9]. Among cognitive deficits, subjective memory complaints are common in the aging population with changes in episodic memory being some of the earliest cognitive changes observed in older adults [10,11,12]. In an effort to more clearly define the role that DHA/EPA supplementation may play in specific aspects of memory function and to better understand responsiveness to DHA/EPA supplementation with aging, the current meta-analysis uniquely categorized results of memory tests from clinical trials into individual memory types, i.e. episodic, semantic, and working memory, and included results from trials of healthy adults (18 years and older) with or without MMC. The current meta-analysis was designed to determine the effect of supplementation of DHA alone, or in combination with EPA, on specific memory functions in healthy adults participating in clinical trials. A secondary objective was to systematically review the observational epidemiologic literature on DHA/EPA and memory outcomes to date in healthy adults.

## Methods

### Information Sources

To identify relevant studies, a comprehensive literature search was conducted by the University of Colorado Denver Health Science Library using two scientific literature databases (Ovid/Medline and Embase) through January 2013. Supplementary literature searches included examining the reference lists of all relevant studies, pertinent review articles, and meta-analyses. Included studies published after the date of literature search through the date of meta-analysis (July 2013) were identified via publication alerts.

### Search Strategy

Relevant terms representing EPA and DHA, as well as their dietary sources, and memory were used for each database searched. Spellings of search terms depended on whether U.S. or European databases were being searched. When appropriate, subject headings were exploded and terms truncated (See Supporting Information—S1 Search Strategy).

### Eligibility Criteria

Clinical trials and observational, epidemiologic studies describing the effect of DHA intake, alone or in combination with EPA, from conventional or fortified sources (foods or dietary supplements) on memory outcomes in healthy adults were considered for review. Eligible studies were conducted in adults residing in the community at baseline with or without MMC. Studies were excluded if: they failed to report memory outcomes, reported fish intake only (i.e., no DHA/EPA levels in diet or blood), studied subjects with current diagnosis of Alzheimer’s disease, dementia, vascular dementia, stroke, head injury, substance abuse, metabolic disturbance, depression, behavioural, or neurologic disorder; enrolled subjects with group mean baseline MMSE scores <24 or reported greater than 10% use of psychotropic, anti-depressant, stimulant, or drugs approved for Alzheimer’s/dementia treatment in the study sample. Studies published in a language other than English were excluded if translation was unavailable. Letters, case reports, position statements, conference proceedings, prevalence surveys, reviews, in vitro studies and studies in animals were excluded. Studies that did not specify the amount or dose of DHA or DHA/EPA supplemented or where DHA/EPA was not evaluated independent of another active, e.g. DHA combined with vitamins/minerals without a DHA or DHA/EPA alone arm, were also excluded.

### Study Selection

Level I screening included a review of all titles and/or abstracts compared to eligibility criteria. Full-text publications of any studies not eliminated at Level I were retrieved for complete review at Level II screening. All search results were screened by two individuals with approximately 95% agreement regarding included and excluded studies. Differences were resolved by discussion and consultation with a third researcher as needed.

### Data Collection Process

A data extraction sheet was created in Excel to capture all data of interest from intervention trials. One independent extractor completed data extraction for all studies, one review author checked text entries, and one independent quality control person checked all numeric outcome data. Study authors (n = 12) were contacted via email to collect results mentioned but not reported, raw data when only adjusted or converted means were presented, and to verify testing methods. Over 80% of authors contacted responded with requested information. Data from observational trials were summarized in narrative format and were not subject to quantitative analysis.

### Data Items

The following list of data items (not exhaustive) were extracted from published intervention trials: (1) study identification details (including study first author; year of study publication; title of publication [1^st^ five words]; country where study was conducted; region of country where study was conducted; study type (RCT or OBS); double-blind [yes or no]; (2) subject baseline demographics (including baseline cognitive status [No cognitive complaints, mild memory complaints]; global dementia score; family history of dementia [%]; APO ε4 status [% negative]; age range; MMSE scores; years of education; distribution of male and female subjects; (3) intervention details (including control product used [name and dosage]; treatment product used [name and dosage]; dose of EPA in treatment [g]; dose of DHA in treatment [g]; dose of EPA+DHA in treatment [g]; food or dietary supplement delivery; duration [days]); (4) dietary details (including baseline diet assessment method, baseline diet information); (5) data details (including number of completed subjects in each group; number of enrolled subjects; name of memory outcome [e.g. immediate recall; verbal fluency, etc.]; (6) outcome assessment method; (7) outcome unit as reported by author; (8) standard deviations [recorded if reported by author or calculated from available data]; (9) pre and post-study memory outcome means. Few studies reported data for global dementia score, family history of dementia and APO ε4 status. More items were collected/calculated than ultimately used in final statistical analysis.

### Memory Tests

Results of neuropsychological tests as reported by study authors were classified into memory types (episodic, semantic, working) and verified by an independent cognition expert. For example, tests of immediate and delayed word list recall, word recognition, story recall, picture recall, the Rey Complex Figure Test, CANTAB PAL, and verbal recognition memory were all classified as tests of episodic memory (See Supporting Information—S1 Table).

Data reported for all outcomes of interest were utilized. In cases where only % accuracy data was reported, the proportion of correct responses was multiplied by the number of test items to obtain the mean and corresponding standard deviation (SD) values.

In some publications authors report individual as well as memory composite score means. Initial extraction collected all available data. However, analyzing both the individual test means and the composite means for a study would double-count the same results data from one study. Data for overlapping composites was not subject to further analysis in favor of keeping individual test means for meta-analysis.

### Summary Measures and Results Synthesis

Effect sizes were based on group mean differences (post-study minus pre-study test scores) and corresponding standard deviations (SDs) between the treatment group and the control (placebo) group. When SDs were not reported, methods described in the Cochrane Handbook for Systematic Reviews of Interventions [13] were relied upon to calculate or estimate SDs from other statistics provided in the published paper (e.g., SDs were calculated from standard errors or confidence intervals) and subsequently recorded in the extraction sheet as calculated values. In cases where means were not reported (i.e. % accuracy data), the proportion of correct responses was multiplied by the number of test items to obtain the mean and corresponding SDs. In some publications where authors reported individual as well as memory composite score means, only individual memory outcome scores were further analyzed.

Effect sizes between treatment and placebo groups for continuous memory outcomes were calculated based on raw group differences as well as standardized group differences using Hedge’s g statistics [14]. This approach permits combining multiple memory-related tests for which different scales were used to measure scores and has been used in a similar meta-analysis [8]. Random-effects meta-analysis models were used to generate between and within group effect sizes.

*A priori* analyses were defined for within and between groups. Macro-level models included data on all subjects, regardless of baseline cognitive status or age, at all dose levels using the longest duration of exposure for each memory type (i.e. episodic, sematic, or working). For each memory type, *a priori* sub-group analyses were conducted for subjects with no cognitive complaints (NCC) vs. those with MMC, age above or below 45 years, and DHA/EPA doses above or below 1 g/day. For studies reporting immediate recall, additional models were considered, including one regardless of cognitive status at baseline and one stratified by NCC vs. MMC. The weight of each study in the meta-analysis was based on the inverse of the variance, a measure that accounts for the sample size within each group. Additional *post-hoc* subgroup analyses were used to complete a comprehensive examination of DHA dose-response to discern any potential pattern or threshold of effect of dose. Heterogeneity was evaluated using the Cochran’s Q statistic and the I^2^ statistic.

### Risk of bias

For RCTs included in the meta-analysis, risk of individual study bias was assessed using the Cochrane risk of bias assessment tool, version 5.1.0. Publication bias on the primary meta-analyses models were evaluated visually using funnel plots and statistically using Egger’s regression techniques, and the Trim and Fill method. All analyses were conducted using Comprehensive Meta-Analyses software, version 2 (Biostat, Englewood, NJ).

## Results

### Search Results

**Fig 1:**
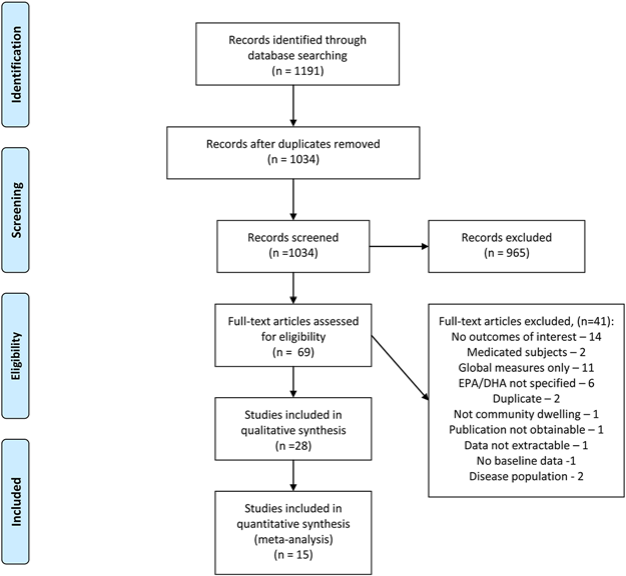
Flow Diagram of Study Selection.

The original search yielded 1191 references, of which 965 were excluded based on initial (Level I) screening of abstracts and/or titles (Fig. 1). The most common reasons for exclusion of trials at Level I screening were irrelevant subject matter or narrative review without original data. Full-text publications of 69 studies were retrieved for complete full-text review at Level II. Citations for studies excluded at Level II with reason for exclusion are listed in Appendix II. Most studies were excluded for failing to provide a memory-related outcome (no outcomes of interest) or only reporting global cognitive scores (global measures only). Of the excluded observational epidemiologic studies, the majority did not estimate DHA/EPA dietary intakes (DHA/EPA not specified). Although some trials and observational studies had more than one reason for exclusion, each study was classified into only one exclusion category to avoid counting a study multiple times. A total of 28 studies (intervention and observational) were included in the qualitative aspect of this review and 15 intervention studies (62 data points) were included in the meta-analysis.

### Study Characteristics

The main study characteristics of the 15 intervention trials included in the meta-analysis are summarized in Table 1. Richter et al. [22] met all inclusion criteria, and is summarized in Table 1, but these data were not included in the between group meta-analysis because the study was not placebo-controlled. These data were, however, included in the within-group meta-analysis. The majority of studies were conducted in subjects without cognitive complaints (n = 9) and ranged from 28–730 days in duration, averaging 4–6 months. Most studies were conducted with subjects ≥ 45 years of age (n = 11) and exclusively with or included an arm with a DHA-rich source of DHA/EPA providing up to 1.55 g DHA per day (n = 12). Sample sizes varied, but most (n = 8) enrolled at least 100 subjects.

**Table 1:** Characteristics of Human Intervention Trials included in Meta-analysis.

Study	Country	Baseline Cognitive Status^a^	Total Subjects Enrolled	Age Range (y)	Duration (days)	TRT^b^	DHA/EPA (g)	DHA (g)	EPA (g)
Benton, 2013 [15]	UK	NCC	285	18–25	50	DHA algal oil	0.4	0.4	0
Dangour, 2010 [16]	England and Wales	NCC	867	70–79	730	Fish Oil	0.7	0.5	0.2
Karr, 2012 [17]	US	NCC	43	18–25	28	Fish Oil	1.2	0.48	0.72
Kotani, 2006 [18]	Japan	MMC	21	>60	90	Aravita dietary supple-ment	0.12	0.12	NR
Lee, 2013 [19]	Malaysia	MMC	36	>60	365	DHA-rich fish oil	1.75	1.3	0.45
Jackson DHA, 2012 [20]	UK	NCC	159	18–35	84	DHA-rich fish oil	0.54	0.45	0.09
Jackson EPA, 2012 [20]	UK	NCC	159	18–35	84	Fish oil	0.5	0.2	0.3
Johnson, 2008 [21]	US	NCC	57	60–80	120	DHA Algal Oil	0.8	0.8	NR
Richter, 2010 [22]	Israel	MMC	8	>60	42	PS-DHA	.037	0.025	0.012
Sinn EPA, 2011 [23]	Australia	MMC	54	>65	180	EPA-rich fish oil	1.83	0.16	1.67
Sinn DHA, 2011 [23]	Australia	MMC	54	>65	180	DHA-rich fish oil	1.95	1.55	0.4
Stonehouse, 2013 [24]	New Zealand	NCC	228	18–45	180	DHA-rich fish oil	1.33	1.16	0.17
Stough, 2012 [25]	UK	NCC	112	45–80	90	DHA-rich fish oil	0.312	0.25	0.06
Vakapova, 2010 [26]	Israel	MMC	157	50–90	105	PS-DHA	0.079	0.06	0.019
Van de Rest < 1g, 2008 [27]	Netherlands	NCC	302	>65	182	Fish oil	0.402	0.176	0.226
Van de Rest >1g, 2008 [27]	Netherlands	NCC	302	>65	182	Fish oil	1.94	0.847	1.093
Witte, 2014 [28]	Germany	NCC	40	50–75	182	Fish oil	2.2	0.88	1.32
Yurko-Mauro, 2010 [29]	US	MMC	485	>55	168	DHA-S Algal Oil	0.9	0.9	≤1%

^a^NCC—no cognitive complaints; MMC—mild memory complaint;

^b^TRT—treatment; DHA—docosahexaenoic acid; ARA—archidonic acid; EPA—eicosapentaenoic acid; PS-DHA—phosphatidylserine-docosahexaenoic acid;

The main study characteristics of the 13 observational studies reviewed are summarized in S2 Table (See Supporting Information). Most (n = 7) were prospective cohort studies of community dwelling individuals (20–70 years old) with no cognitive complaints at baseline. Studies (n = 10) primarily examined the association between DHA/EPA blood levels and cognitive outcomes although some reported the association between cognition and daily DHA/EPA dietary intake. Studies were primarily conducted in European Union countries (n = 9) followed by the U.S. (n = 3) and Australia (n = 1).

### Meta-Analysis Results

The majority of included studies were of low (n = 8) to moderate risk (n = 4) of bias. Three studies were of high risk of bias of which one [22] was only included in within-group analyses due to single blind study design. Of the remaining two high risk studies [15,18], only one [18] contributed data to episodic memory outcomes. A total of 62 data points were included in the overall meta-analysis model for episodic memory. The number of data points exceeds the number of RCTs because most studies reported relevant data for multiple dose levels or memory tests. The overall model included all dose levels and all subjects, regardless of cognitive status at baseline (See Supporting Information—S1 Fig.). The overall model and the analysis of episodic memory in response to DHA/EPA supplementation among adults with no cognitive complaints at baseline was not significant (Table 2). Based on visual inspection of the funnel plot, publication bias was not apparent. Furthermore, statistical tests did not indicate the presence of publication bias (Egger’s regression p-value > 0.05, no studies were imputed on either side of the effect size based on the trim and fill method).

**Table 2:** Summary of DHA/EPA^a^ Supplementation and Episodic Memory Outcomes in Adults.

Between Group	# Data Points	WGMD^b^	Z-score	p-Value Z-score	p-H	Hedge's g WGMD	Hedge's Z-score	p-Value Hedge's Z	p-H^c^
Overall	62	0.08	1.777	0.076	0.478	0.03	1.647	0.1	0.524
All NCC^d^	44	0.029	0.511	0.609	0.842	0.000	0.012	0.99	0.853
All MMC^e^	18	**0.234**	**1.932**	**0.053**	0.093	**0.114**	**2.86**	**0.004**	0.311
Age ≤45 years	19	-0.006	-0.044	0.965	0.149	-0.012	-0.251	0.802	0.165
Age >45 years	43	0.092	1.861	0.063	0.715	0.039	1.897	0.058	0.767
DHA+EPA ≤1g	43	0.054	1.090	0.277	0.428	0.018	0.896	0.37	0.442
DHA+EPA >1g	19	0.213	1.810	0.070	0.563	**0.096**	**2.057**	**0.04**	0.686
Immediate Recall—all	20	0.094	1.130	0.257	0.570	0.010	0.328	0.743	0.512
Immediate Recall—NCC	13	-0.063	-0.557	0.577	0.830	-0.029	-0.861	0.389	0.864
Immediate Recall—MMC	7	**0.274**	**2.250**	**0.024**	0.448	**0.173**	**2.501**	**0.012**	0.627
**Within Group**	67	**0.844**	**7.180**	**0.000**	0.000	**0.224**	**5.903**	**0.000**	0.000
All NCC	44	**0.534**	**4.871**	**0.000**	0.000	**0.189**	**4.757**	**0.000**	0.000
All MMC	23	**1.967**	**5.989**	**0.000**	0.000	**0.338**	**3.784**	**0.000**	0.000
Age ≤45 years	19	**0.67**	**2.949**	**0.003**	0.000	**0.213**	**2.922**	**0.003**	0.000
Age >45 years	48	**0.911**	**6.55**	**0.000**	0.000	**0.226**	**5.125**	**0.000**	0.000
DHA+EPA Intakes ≤1g	48	**0.719**	**5.493**	**0.000**	0.000	**0.182**	**4.080**	**0.000**	0.000
DHA+EPA Intakes >1g	19	**1.362**	**4.935**	**0.000**	0.000	**0.329**	**5.629**	**0.000**	0.000
Immediate Recall—all	21	**1.663**	**5.614**	**0.000**	0.000	**0.299**	**4.523**	**0.000**	0.000
Immediate Recall—NCC	13	**1.078**	**3.734**	**0.000**	0.000	**0.241**	**3.439**	**0.001**	0.000
Immediate Recall—MMC	8	**2.926**	**3.117**	**0.002**	0.000	**0.486**	**2.756**	**0.006**	0.000
**Dose Subgroup analysis**									
DHA <580 mg	34	0.037	0.480	0.631	0.333	0.003	0.136	0.892	0.358
DHA >580 mg	28	0.108	1.846	0.065	0.606	**0.079**	**2.596**	**0.009**	0.819
DHA >580 mg NCC	18	0.063	0.704	0.481	0.982	0.036	0.745	0.456	0.984
DHA >580 mg MMC	10	0.203	1.402	0.161	0.050	**0.117**	**2.345**	**0.019**	0.212
DHA ≤250 mg	18	0.065	0.491	0.623	0.338	0.014	0.288	0.773	0.359
DHA 251–500 mg	16	0.018	0.188	0.851	0.320	-0.001	-0.023	0.981	0.333
DHA 501–999 mg	19	0.096	1.497	0.134	0.844	**0.071**	**2.077**	**0.038**	0.937
DHA 1 g+	9	0.238	1.048	0.295	0.144	0.128	1.592	0.111	0.255

^a^DHA/EPA = docosahexaenoic acid alone or in combination with eicosapentaenoic acid;

^b^WGMD = weighted group mean difference;

^c^p-H = p-value for heterogeneity test;

^d^NCC = subjects without cognitive complaints at baseline;

^e^MMC = subjects with mild memory complaints at baseline. Bold text indicates statistically significant result.

**Fig 2:**
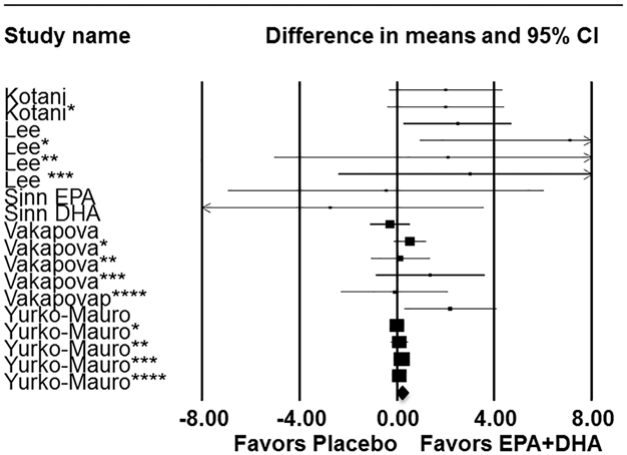
Forest plot with meta-analysis of episodic memory data. Episodic memory data from pertinent human intervention studies (n = 5) of DHA supplementation in adults with mild memory complaints. *Asterisks denote study included more than one test of episodic memory, results for each episodic memory test within a given study represented individually. Summary statistics are as follows z = 1.932 (p = 0.053); Hedge’s g z = 2.86 (p = 0.004).

**Fig 3:**
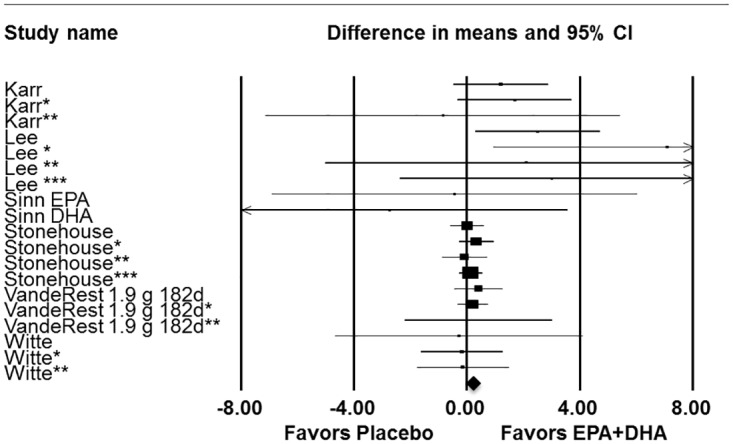
Forest plot with meta-analysis of dose and episodic memory. Episodic memory data from pertinent human intervention studies (n = 6) of DHA supplementation alone or in combination with EPA > 1g in all adults. *Asterisks denote study included more than one test of episodic memory, results for each episodic memory test within a given study represented individually. Summary statistics are as follows z = 1.81 (p = 0.070); Hedge’s g z = 2.05 (p = 0.04).

In the *a priori* meta-analysis model of adults with MMC, episodic memory significantly (P<.004) improved in response to DHA supplementation, alone or in combination with EPA (Fig. 2; Table 2). Regardless of cognitive status, combined DHA/EPA supplementation of > 1 g/day improved episodic memory (Fig. 3; Table 2) and there was a trend (P<.058) for episodic memory improvement in response to supplementation in subjects 45 years or older (Table 2). Based on prior meta-analytical results reported by Mazereeuw and co-workers [8], *a priori* analyses of immediate recall tests were conducted. Immediate recall was significantly (P<.012) improved in response to supplementation in adults with MMC at baseline (Table 2). The effects of DHA/EPA supplementation were relatively consistent within meta-analysis models and in various sub-group analyses as demonstrated by non-significance in tests of heterogeneity. As most studies examined the role of DHA-rich supplements, post-hoc sub-group analyses were conducted to determine if a particular dose of DHA was associated with episodic memory improvement. DHA intake above the mean DHA level studied (580 mg/day) significantly improved episodic memory in all subjects (P<.009) and in subjects with MMC (P<.019) (Table 2). This effect appears to be largely driven by studies providing between 501–999 mg DHA daily (Table 2).

The absolute effect “within” an exposure group is important to determine whether the supplement provides a benefit independent of a comparator. The absolute within group difference was statistically significant for all episodic memory models examined (Table 2). These within group absolute differences substantiate the findings from the between group analyses and support a beneficial effect of DHA/EPA for episodic memory.

Fifteen data points were included in the overall meta-analysis model for semantic memory. No significant effects of DHA/EPA supplementation were found for any of the between group models analyzed (See Supporting Information—S3 Table). However, significant improvements from baseline were observed in the overall within group analyses. The models of semantic memory improvements from baseline in adults older than 45 and those supplemented with more than 1 g DHA+EPA daily were also statistically significant (See Supporting Information—S3 Table). Based on visual inspection of the funnel plot and Egger’s regression test, publication bias was not apparent for studies of semantic memory.

Twenty-one data points were included in the overall meta-analysis model for working memory. No significant effects of DHA/EPA supplementation were found for any of the between group models analyzed (See Supporting Information—S4 Table). Significant improvement from baseline in working memory outcomes (P<.027) was observed in the within group analyses. The models of working memory improvements from baseline in adults older than 45 and those supplemented with more than 1 g DHA+EPA daily were also statistically significant (P<.028; P<.001, respectively). Based on visual inspection of the funnel plot and Egger’s regression test, publication bias was also not apparent for studies of working memory.

### Observational Studies Summary

A complete summary of the results from observational studies is available in Table 3. Only two observational studies [31,36] examined the association between DHA/EPA blood levels and memory outcomes in younger populations. Consistent with the current meta-analysis results for younger adults (i.e., <45 years), De Groot et al. [31] did not find an association between higher DHA status and tests of episodic memory. In contrast, Muldoon et al [36] showed that higher serum phospholipid DHA was associated with better performance on nonverbal reasoning, logical memory, and working memory in healthy middle-aged adults ages 30–54 (mean age = 44.6±6.7). Overall, results from observational studies support a broad role of DHA/EPA status and improved cognitive outcomes in older adults (≥45 years). Most observational studies reporting the association between DHA/EPA blood levels and memory outcomes found improvements between these fatty acids and at least one memory type or other cognitive outcome. Beydoun and co-workers [30] reported a beneficial association between plasma DHA/EPA and semantic memory function. Tan and co-workers [39] reported a beneficial association between erythrocyte DHA and visual but not verbal episodic memory. Titova and co-workers [40] found a positive association between dietary DHA/EPA and composite cognitive scores (including a memory component), and Phillips and co-workers [37] found a significant association between both plasma and DHA/EPA dietary intake and composite memory scores. Four studies found no association between either DHA/EPA blood levels [32,35] or dietary intake [33,34] and memory function in older adults, but reported benefits of higher DHA/EPA status to processing speed [32,33] and subject-reported cognitive complaints [34,35]. Finally, among studies reporting APOE ε4 status, Samieri et al. [38] reported a positive association between plasma DHA and working memory in ApoE4 positive subjects and Whalley and co-workers [42] found higher erythrocyte DHA/EPA levels associated with higher scores on tests of immediate recall in APOE ε4 negative participants [41].

**Table 3:** Summary of Results from Relevant Observational Studies Regarding DHA/EPA Status and Memory Outcomes.

Study	Memory Outcome (Test)	Observation	Conclusion
Beydoun, 2007 [30]	Recent Memory (DWRT)^a^	No effect was observed on delayed word recall among any of the subgroups considered.	“Promoting higher intakes of n-3 HUFAs^b^ in the diet of hypertensive and dyslipidemic persons may have substantial benefits in reducing their risk of cognitive decline in the area of verbal fluency. However, clinical trials are needed to confirm this finding.”
de Groot, 2007 [31]	Memory (Visual Verbal Word Learning Tasks)	WLTtot^c^ (# words)*and WLTdr^d^ (# words) were significantly different between baseline and 22 weeks (P<0.05). Performances on WLTtot (P = 0.002), WLTdr (P = 0.014) were significantly better at 22 week.	“In conclusion, this study provides a preliminary indication that a higher DHA^e^ status might be associated with slower learning curves. However, additional studies are necessary.”
Dullemeijer, 2007 [32]	Memory *Z*-Score (Immediate & delayed recall)	“Participants improved on memory over 3y; the mean (± SD) 3-year change in *z* scores was 0.34 ± 0.73 for memory, because of procedural learning effects.”	In older Dutch adults, higher plasma n-3 PUFA were not associated with less decline in 3-year word fluency
Kalmijn, 2004 [33]	Memory Function *Z*-Scores (Total, maximal, & delayed recall scores for Verbal Learning Test were averaged.)	Adjusted OR* for risk of impaired memory function (lowest 10%) according to one SD increase in fatty fish or fatty acid intake. EPA + DHA 1.01 (0.85–1.20) Fatty Fish 0.95 (0.80–1.13) *OR^h^ (95% CI) adjusted for age, sex, education, alcohol, smoking, & total energy intake	“Fatty fish and marine omega-3 PUFA^i^ consumption was associated with a reduced risk … of impaired cognitive function in this middle-aged population.”
Kesse-Guyot, 2011 [34]	Memory Test (5-word test of immediate & delayed verbal memory including free & cued recall	No significant association between poor scores on the MMSE^l^ and 5-word cognitive tests and intake of fish and related fatty acids.”	“Cognitive complaints, which may be an early indicator of cognitive decline, are less frequent among the elderly who have a high long-chain n-3 acids intake, as assessed 13 years earlier.”
Milte, 2011 [35]	Immediate Memory (RAVLT)^p^	Higher levels of the n-6 PUFAs DGLA, AA, DPA and lower levels of the n-3 PUFAs EPA and DPA were associated with poorer performance on various cognitive assessments and self- reported scales. In addition, lower levels of various n-3 PUFAs were associated with higher levels of n-6 PUFAs, including n-6 DPA.”	“Despite limitations, this study adds to the growing evidence of a possible role of PUFAs in memory problems and mood in the elderly. It points to differences in n-3 and n-6 PUFA status in adults with MCI compared with healthy controls. It also provides evidence of associations between higher n-6 DPA status and poorer cognition, memory and perception of health, which are modulated by depressive symptoms. It suggests that modification of PUFA intakes may positively affect mood and memory in adults with MCI….”
Muldoon, 2010 [36]	General / Episodic Memory & Working Memory (WMS-3)^q^	Higher DHA blood levels were associated with better scores on tests of working memory. In contrast, EPA was marginally associated (P = 0.054) only with working memory.	“The findings in this report suggest that DHA may be the (n-3) fatty acid most closely related to cognitive function.”
Phillips, 2012 [37]	Composite Memory Score	“In this model* plasma PC EPA and DHA, and dietary omega-3 intake score were all positive predictors of the composite memory performance score.” *Controlling for age, years of education, IMDs, and sex.	”These results are consistent with the possibility that omega-3 fatty acid nutrition has an impact on cognitive decline, but could equally be explained by dietary changes that occurred after onset of cognitive decline. It is also possible that the results could be explained by unknown confounding factors.”
Samieri, 2011 [38]	Working Memory (BVRT)^t^	A significant interaction was found between plasma DHA proportion and the ApoE-ε4^u^ on the change of BVRT scores over time (*p* = 0.02). A 1-SD increase in plasma DHA was not associated with the evolution of BVRT performances over time in ApoE-ε4 non-carriers, but strongly related to slower decline on BVRT performances in ApoE-ε4 carriers (*β* = 0.061 (SD = 0.024, *p* = .01).	“Plasma DHA was associated with slower decline on BVRT performances in ApoE- ε 4 carriers only. EPA and DHA may contribute to delaying decline in visual working memory in ApoE- ε 4 carriers. In older depressed subjects, EPA, but not DHA, may slow cognitive decline.”
Tan 2012 [39]	Verbal Memory (LM-d)^v^; Visual Memory (VR-d^w^)	RBC^x^ DHA levels showed a continuous positive association with performance in tests of visual memory (VR-d). There was no statistically significant relationship between RBC DHA and performance on verbal memory (LM-d).	“Lower RBC DHA levels are associated with smaller brain volumes and a ‘vascular’ pattern of cognitive impairment even in persons free of clinical dementia.”
Titova, 2013 [40]	Declarative Memory—7 minute screening (ECR)^y^	The dietary intake of EPA and DHA was positively linked to the 7MS^z^ score (i.e., the total score obtained on 4 cognitive subtests, 1 of which was the ECR). This association remained significant in all models. However, there were no associations between plasma EPA or DHA with the performance on the 7MS estimates.	“Study results provide a potential link between diets rich in EPA & DHA & enhanced mental health in the elderly.”
Whalley, 2004 [41]	Immediate Memory (AVLT)^aa^	Pearson’s correlation coefficients between AVLT scores and log-transformed RBC LCPUFAs^bb^ content was not statistically significant.	Total RBC n-3 FA & ratio of DHA to AA was associated with better cognitive function in late life before and after adjustment for childhood IQ.”If associations with n-3 content are causal, optimization of n-3 and n-6 fatty acid intakes could improve retention of cognitive function in old age.”
Whalley, 2008 [42]	Immediate Memory (AVLT)	DHA was associated with significantly (*P* <0.001) higher scores on AVLT. DHA also had a differential effect over time (*P* <0.001): AVLT was significantly (*P* <0.001) lower at wave 1 and wave 2 than at wave 3.	Cognitive benefits were associated with higher erythrocyte n-3 PUFA content but were significant only in the absence of the APOE ε4 allele. “These data are evidence of a gene X environment interaction for cognitive aging. They are relevant to the analysis of trials of n-3 PUFA supplements in cognitive aging and dementia prevention….”

^a^DWRT = Delayed Word Recall Test;

^b^ HUFAs = highly unsaturated fatty acids;

^c^ WLTtot = Word Learning Task total;

^d^ WLTdr = Word Learning Tasks delayed recall;

^e^ DHA = Docosahexaenoic Acid;

^f^ SD = Standard Deviation;

^g^ EPA = Eicosapentaenoic Acid;

^h^ OR = Odds Ratio

^i^ PUFA = Polyunsaturated Fatty Acids;

^j^BMI = body mass index;

^k^ CES-D = Center for Epidemiologic Studies Depression Scale (CES-D);

^l^ MMSE = Mini-Mental State Examination;

^m^ MCI = mild cognitive impairment;

^n^DGLA = digamma linoleic acid;

^o^; AA—Arachidonic Acid;

^p^ RAVLT = Rey Auditory Verbal Learning Test;

^q^ WMS-3 = Wechsler Memory Scale, 3^rd^ ed.;

^r^ PC = Phosphatidylcholine;

^s^ IMD = Index of Multiple Deprivation;

^t^ BVRT = Benton Visual Retention Test;

^u^ APOE ε4 = Apolipoprotein E Allele 4;

^v^ LM-d = Logical Memory test—delayed;

^w^ VR-d = Visual Reproduction test delayed recall;

^x^ RBC = Red Blood Cell;

^y^ ECR = Enhanced Cued Recall;

^x^7MS = 7-Minute Scoring of the ECR;

^aa^ AVLT = Rey Auditory Verbal Learning Test;

^bb^ LCPUFA = Long-chain Polyunsaturated Fatty Acids;

## Discussion

Memory is divided into two general types, declarative and non-declarative. Declarative memory relates to the conscious recollection of facts and events and can be further sub-divided into episodic and semantic [43]. Episodic memory is memory for personally experienced events that occur at a specific place and time and is measured by memory of stories, word lists, or figures [43]. Episodic memory typically declines throughout life and is consistent with normal, healthy aging decline [43]. Aging adults are concerned with memory loss, even more so than with cardiovascular health or physical activity [44]. The current meta-analysis results with DHA suggest a benefit of supplementation in improving episodic memory function in healthy adults with MMC and are consistent with those of Mazereeuw and co-workers [8] for improvements in the immediate recall sub-category of episodic memory. The strength and uniqueness of the current meta-analysis approach is that it allows demonstration of DHA/EPA improvements across a variety of individual memory tests within the category of episodic memory not otherwise noticeable in a composite of memory outcomes. The studies included in this meta-analysis varied in sample size and demographics (e.g. age, gender, education) as well as duration of supplementation, yet the effect of DHA/EPA supplementation across these studies was significant. Examining the data in this manner also allowed for the observation of additional relationships, in particular, the role of DHA/EPA dose in memory improvement. While Mazereeuw et al. [8] did not find an association of dose on the treatment effect size for a single test of episodic memory, i.e. immediate recall, the current analysis found that multiple tests for episodic memory, regardless of cognitive status (MMC and NCC combined, n = 19 data points), demonstrated an improvement in performance from at least 1 g/d of DHA+EPA. Data showed that this benefit is apparently driven by DHA, in particular, at a level between 501–999 mg/day. Data were likely insufficient to evaluate doses of DHA >1 g, as only 9 data points were available from studies providing this dose range. Discrepancies between our meta-analysis findings and 2 recent meta-analyses are likely due to differences in designs and criteria for combining studies on n-3 LCPUFA and cognition. Jiao and co-workers [45] examined studies across wide age ranges, i.e. infancy to elderly and did not provide appropriate sub-group analyses by age or dose. This approach is problematic as cognitive assessment is dependent upon brain development milestones in the young and is not pertinent to older adults, and use of such wide doses within a single analysis makes meaningful interpretation of results difficult. The meta-analysis by Abubakari and co-workers [7] combined studies of healthy adults with disease populations (e.g. AD, depression, schizophrenia) and thus included a large array of cognitive tests. Heterogeneity of baseline cognitive function across these populations and varied sensitivity of assessments may have obscured important between group differences for DHA/EPA supplementation versus placebo.

The direct role of diet in the modulation of DHA content in specific regions of the brain at key points during the lifecycle is likely an important contributor to memory development and continued normal memory function. Evidence from human autopsy supports a rapid accretion of DHA throughout early development [46, 47] to 18 years of age, then a continuous but more gradual accumulation of DHA in cerebral cortex fatty acids in adulthood [46,47]. Specifically, evidence supports roughly 10% concentration of DHA in normal human cerebral cortex tissue at birth that increases progressively to about 27% by 16 years of age [46,48]. The hippocampus, in particular, is essential for memory function. Dietary deficiency and repletion of DHA in the cerebral cortex of neonates and adults (both young and old), particularly in the frontal cortex, and hippocampus has been demonstrated in rodents [49, 50] and non-human primates [51]. However, in humans both cerebral cortex and hippocampal volume have been shown to decrease with age and memory loss, as recently demonstrated [52]. In a cohort of healthy, MCI and AD subjects combined (with or without ApoE4^+^ genotype), significant decreases in hippocampal and cerebral cortex grey matter volume were found [52]. Yet among fish oil supplement users in this cohort, less cerebral cortex and hippocampal atrophy was shown compared to non-users and this relationship was particularly significant for the ApoE4^-^ genotype. Fish oil supplement users with normal cognitive function and those with the ApoE4^-^ genotype also showed significant associations with global cognitive tests (ADAS-Cog and MMSE) at any point in time during DHA/EPA use, suggesting that maintaining brain DHA/EPA content throughout adulthood may help prevent functional cognitive declines with aging.

From a mechanistic view, in early life, animal studies support a direct role of DHA in neurogenesis, synaptogenesis, and myelination [50]. DHA modulation of hippocampal neurons, both *in vitro* and *in vivo* from neonatal animals, supports its role in growth and maturation that likely contributes to improvements in performance of memory-related tasks such as the Morris water maze [50]. Recent recognition of the existence of adult neural stem cells and the ability of these cells to support neurogenesis in adulthood supports the possibility that DHA plays a continuous role in hippocampal maintenance that supports memory function throughout life [53]. DHA and EPA have both been found to directly impact neuronal differentiation in cultured rat neural stem cells through separate but complementary mechanisms [54]. Aged animal models (e.g. SAMP8 mouse, and aged rats) show depleted levels of DHA in brain whereas DHA supplementation restores not only brain phospholipid containing DHA levels, but improves learning and memory retention on tasks such as Morris water maze, radial arm maze, and T-maze avoidance [55,56, 57]. These preclinical models demonstrate the important role that DHA serves in not only brain development but also in maintaining cognitive function in aging.

In our meta-analysis, lack of an apparent relationship between episodic memory and DHA/EPA supplementation among adults with no cognitive complaints at baseline may be related to several factors. It has been hypothesized that the cognitive benefits of n-3 LCPUFA supplementation are not detectable in people with a high cognitive reserve or that the neuroprotection supported by DHA requires treatment in proximity of cognitive decline to detect a benefit [4]. Variability in subjects’ cognitive function and a lack of consistent screening tests with cut-offs for baseline memory scores may also contribute to null findings in some clinical studies of n-3 LCPUFA supplementation of older adults. Disparities in dietary intake and subsequent blood levels of n-3 LCPUFA at baseline may also contribute to limited findings. Observational data suggests that risk of cognitive decline may be greatest in populations with lower n-3 LCPUFA intakes [3,4].

In contrast to intervention trials, associations reported in our systematic review of observational trials were not limited to episodic memory improvements but included benefits to working, semantic, and composite memory scores as well. An association between DHA/EPA and semantic and working memory likely reflects the long-term follow-up of subjects in observational trials. Declines in semantic and working memory reflect later stages of cognitive decline [12] and likely require study in excess of the average 4–6 months of intervention studies reviewed herein to recognize benefits.

### Limitations

The quality of the included intervention studies was reasonable. Studies rated as having a high risk of bias were few but may have affected the reliability of the meta-analysis. Only 1 [18] of 3 [15,18,22] studies with a high risk of bias contributed episodic memory data. The combining of various tests into specific memory types was completed by a third party cognition expert but all experts may not agree upon which tests measure which type of memory. Finally, while every effort was made to only include studies of healthy subjects, either with no or only mild cognitive complaints, it is possible that some study populations may have included subjects with undiagnosed pre-dementia. Insufficient data are available from current RCTs to assess the interaction between gender, age, and APOE status on cognitive outcomes following DHA/EPA supplementation. It has been proposed by Stonehouse that, “…if dimorphisms exist for any of these factors, potential effects may be diluted or cancelled out resulting in biased results” [58].

## Conclusions

DHA supplementation, alone or in combination with EPA, is associated with improved episodic memory in adults with mild memory complaints. The meta-analytic results demonstrate a significant impact on age-associated memory loss which is a major health concern of older adults. Additional long-term intervention studies examining the role that DHA/EPA may play in semantic and working memory outcomes in aging individuals are needed. Observational data support a broad role of DHA/EPA intake and its positive effects on memory and cognition in older adults.

## Supporting Information

S1 Checklist Completed PRISMA Checklist. (DOC)

S1 Fig Forest plot with meta-analysis of episodic memory in response to DHA supplementation in all adults. (PPTX)

S1 Search Strategy Electronic Search Strategy for Ovid Medline. (DOCX)

S1 Table Memory Types Assigned to Outcomes in Published Studies. (DOCX)

S2 Table Characteristics of Observational Studies Reporting DHA/EPA Status and Memory Outcomes. (DOCX)

S3 Table Summary of DHA/EPA Supplementation and Semantic Memory Outcomes in Adults. (DOCX)

S4 Table Summary of DHA/EPA Supplementation and Working Memory Outcomes in Adults. (DOCX)
