# Supplementary material for: Docosahexaenoic Acid and Adult Memory: A Systematic Review and Meta-Analysis
Source: PLoS One. 2015 Mar 18;10(3):e0120391. doi: 10.1371/journal.pone.0120391 (PMC4364972; doi:10.1371/journal.pone.0120391)
Supplement: S1 Fig — (PPTX) [file pone.0120391.s002.pptx]

## Slide 1
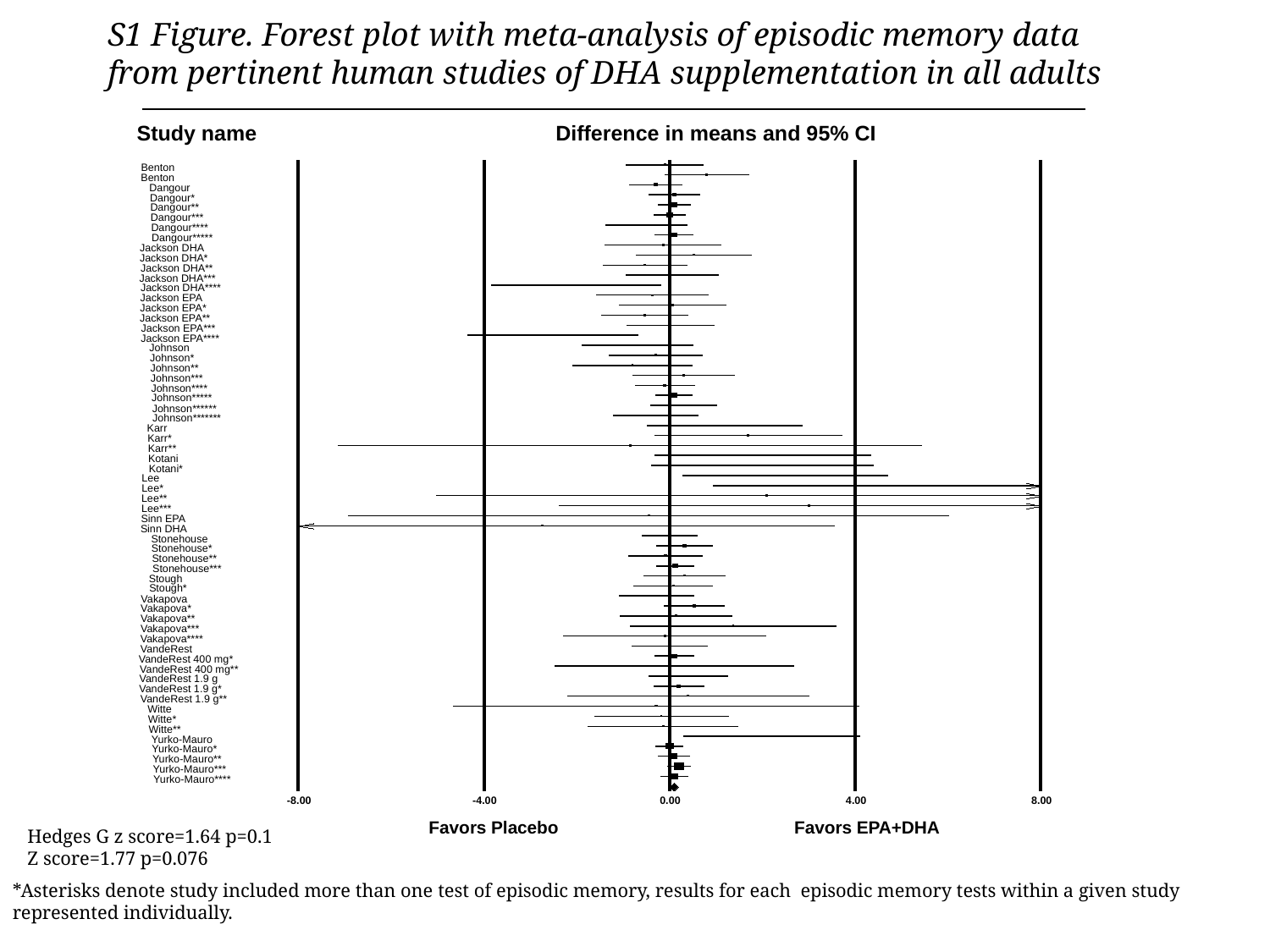

S1 Figure. Forest plot with meta-analysis of episodic memory data from pertinent human studies of DHA supplementation in all adults
Study name
Difference in means and 95% CI
Benton
Benton
Dangour
Dangour*
Dangour**
Dangour***
Dangour****
Dangour*****
Jackson DHA
Jackson DHA*
Jackson DHA**
Jackson DHA***
Jackson DHA****
Jackson EPA
Jackson EPA*
Jackson EPA**
Jackson EPA***
Jackson EPA****
Johnson
Johnson*
Johnson**
Johnson***
Johnson****
Johnson*****
Johnson******
Johnson*******
Karr
Karr*
Karr**
Kotani
Kotani*
Lee
Lee*
Lee**
Lee***
Sinn EPA
Sinn DHA
Stonehouse
Stonehouse*
Stonehouse**
Stonehouse***
Stough
Stough*
Vakapova
Vakapova*
Vakapova**
Vakapova***
Vakapova****
VandeRest
VandeRest 400 mg*
VandeRest 400 mg**
VandeRest 1.9 g
VandeRest 1.9 g*
VandeRest 1.9 g**
Witte
Witte*
Witte**
Yurko-Mauro
Yurko-Mauro*
Yurko-Mauro**
Yurko-Mauro***
Yurko-Mauro****
-8.00
-4.00
0.00
4.00
8.00
Favors Placebo
Favors EPA+DHA
Hedges G z score=1.64 p=0.1
Z score=1.77 p=0.076
*Asterisks denote study included more than one test of episodic memory, results for each episodic memory tests within a given study represented individually.
