# Supplementary material for: Docosahexaenoic Acid and Adult Memory: A Systematic Review and Meta-Analysis
Source: PLoS One. 2015 Mar 18;10(3):e0120391. doi: 10.1371/journal.pone.0120391 (PMC4364972; doi:10.1371/journal.pone.0120391)
Supplement: S1 Search Strategy — (DOCX) [file pone.0120391.s003.docx]

Search Strategy S1. Electronic Search Strategy for Ovid Medline

Database: Ovid MEDLINE(R) In-Process & Other Non-Indexed Citations, Ovid MEDLINE(R) Daily and Ovid MEDLINE(R) <1946 to Present>

Search Strategy:

--------------------------------------------------------------------------------

1 Eicosapentaenoic Acid/ or eicosapentaenoic.mp. (6862)

2 (epa and fatty acid*).mp. (3545)

3 1 or 2 (7428)

4 Docosahexaenoic Acids/ or docosahexaenoic.mp. (8521)

5 (dha and fatty acid*).mp. (4518)

6 4 or 5 (9138)

7 exp Fish Oils/ (16998)

8 (fatty acid* adj5 (omega 3 or omega-3 or omega3 or n3 or n-3)).mp. (13986)

9 (fish adj1 (intake or consum* or diet)).mp. (2664)

10 7 or 8 or 9 (22946)

11 3 or 6 or 10 (25366)

12 [] (0)

13 [] (0)

14 11 (25366)

15 adult/ or aged/ or "aged, 80 and over"/ or frail elderly/ or middle aged/ or young adult/ (5164787)

16 adolescent/ or child/ or child, preschool/ or infant/ or infant, newborn/ or infant, low birth weight/ or infant, small for gestational age/ or infant, very low birth weight/ or infant, extremely low birth weight/ or infant, postmature/ or infant, premature/ or infant, extremely premature/ (2684747)

17 15 and 16 (1252492)

18 16 not 17 (1432255)

19 14 not 18 (23914)

20 [] (0)

21 [] (0)

22 [] (0)

23 [] (0)

24 19 (23914)

25 limit 24 to humans (13164)

26 human*.mp. (12906159)

27 24 and 26 (14023)

28 25 or 27 (14023)

29 [] (0)

30 [] (0)

31 [] (0)

32 [] (0)

33 [] (0)

34 28 (14023)

35 exp treatment outcome/ (569247)

36 clinical trial.mp. (548827)

37 clinical trials.mp. (273480)

38 (clinical adj trial$1).mp. (731814)

39 random$.mp. (794054)

40 exp research design/ (311217)

41 comparative study/ or placebos/ (1624637)

42 placebo$.mp. (158366)

43 multicenter study.mp. (153329)

44 double blind method/ (117247)

45 (single blind$3 or double blind$3 or triple blind$3).mp. (163981)

46 exp "epidemiologic research design"/ (765476)

47 clinical protocols/ (18341)

48 feasibility studies/ (37722)

49 practice guideline.mp. (19097)

50 36 or 39 or 44 (1090308)

51 or/35-49 (3479353)

52 34 and 51 (5010)

53 limit 34 to (clinical trial, all or clinical trial, phase i or clinical trial, phase ii or clinical trial, phase iii or clinical trial, phase iv or clinical trial or controlled clinical trial or randomized controlled trial) (2306)

54 52 or 53 (5010)

55 [] (0)

56 [] (0)

57 [] (0)

58 [] (0)

59 54 (5010)

60 cognition disorders/ or auditory perceptual disorders/ or huntington disease/ or mild cognitive impairment/ (54125)

61 cognition/ or awareness/ or cognitive dissonance/ or cognitive reserve/ or comprehension/ or consciousness/ or imagination/ or dreams/ or fantasy/ or intuition/ (98005)

62 exp Learning/ or learning.mp. (350147)

63 ((age adj1 (relate* or associate*)) and ((memory or cognit*) adj1 (decline or impair*))).mp. (2304)

64 (((memory or cognit*) adj1 (decline or impair*)) not (alzheimer or dementia or stroke)).mp. (24636)

65 (mild adj cognitive adj impairment).mp. (5711)

66 exp memory/ or memory.mp. (177882)

67 exp Memory Disorders/ (20272)

68 60 or 61 or 62 or 63 or 64 or 65 or 66 or 67 (530686)

**69 59 and 68 (174)**

70 [] (0)

71 [] (0)

72 [] (0)

73 [] (0)

74 69 (176)

75 exp animal experimentation/ or exp models, animal/ (384760)

76 74 not 75 (171)

77 [] (0)

78 [] (0)

79 34 [no clinical trails parsing] (14023)

80 68 and 79 [cognitive disorders parsing] (408)

81 80 not 75 [no animal experimentation parsing] (388)

**82 81 not 69 [previous set with clinical trials removed] (217)**
