# Supplementary material for: Docosahexaenoic Acid and Adult Memory: A Systematic Review and Meta-Analysis
Source: PLoS One. 2015 Mar 18;10(3):e0120391. doi: 10.1371/journal.pone.0120391 (PMC4364972; doi:10.1371/journal.pone.0120391)
Supplement: S1 Table — (DOCX) [file pone.0120391.s004.docx]

| **S1 Table. Memory Types Assigned to Outcomes in Published Studies** | | |
| --- | --- | --- |
| **Outcome** | **Outcome assessment method** | **Memory Type** |
| immediate recall | Auditory verbal learning test | episodic |
| delayed recall | Auditory verbal learning test | episodic |
| word recognition | Auditory verbal learning test | episodic |
| immediate recall | word list recall | episodic |
| delayed recall | word list recall | episodic |
| immediate recall | story recall | episodic |
| delayed recall | story recall, delayed | episodic |
| spatial memory immediate | location recall | episodic |
| spatial memory delayed | location recall, delayed | episodic |
| immediate recall | California Verbal Learning Test | episodic |
| delayed recall | California Verbal Learning Test | episodic |
| word recognition | delayed word recognition | episodic |
| picture recognition | delayed picture recognition | episodic |
| names to faces recall | 12 photos 4 diff 1st and last names | episodic |
| Shopping list memory test | Shopping list task | episodic |
| shopping list memory test delayed | Shopping list task | episodic |
| word list memory test | word list memory test computer version | episodic |
| word list memory test delayed | word list memory test computer version | episodic |
| MIR Apartment test delayed | MIR apartment test | episodic |
| MIR Apartment test location | MIR apartment test | episodic |
| immediate recall | Rey Auditory Verbal Learning | episodic |
| delayed recall | Rey Auditory Verbal Learning | episodic |
| immediate recall | Japanese Repeatable Battery for the Assessment of Neuropsychological Status | episodic |
| delayed recall | Japanese Repeatable Battery for the Assessment of Neuropsychological Status | episodic |
| visual reproduction I | Weschler memory scale -revised | episodic |
| visual reproduction II | Weschler memory scale -revised | episodic |
| immediate recall | Cognitive Drug Research computerized assessment system | episodic |
| delayed recall | Cognitive Drug Research computerized assessment system | episodic |
| decay immediate to delayed word recall | Cognitive Drug Research computerized assessment system | episodic |
| word recognition | Cognitive Drug Research computerized assessment system | episodic |
| picture recognition | Cognitive Drug Research computerized assessment system | episodic |
| immediate recall | Computerized mental performance assessment system | episodic |
| delayed recall | Computerized mental performance assessment system | episodic |
| delayed word recognition | Computerized mental performance assessment system | episodic |
| delayed picture recognition | Computerized mental performance assessment system | episodic |
| immediate recall | Rey Complex Figure Test | episodic |
| delayed recall | Rey Complex Figure Test | episodic |
| delayed recall | 15 word learning | episodic |
| word recognition | 15 word learning | episodic |
| immediate recall | 15 word learning | episodic |
| CANTAB PAL | Cambridge Neuropsychological Test Automated Battery | episodic |
| Pattern Recognition Memory | Cambridge Neuropsychological Test Automated Battery | episodic |
| Verbal Recognition Memory, Free Recall | Cambridge Neuropsychological Test Automated Battery | episodic |
| Verbal Recognition Memory, Immediate Recall | Cambridge Neuropsychological Test Automated Battery | episodic |
| Verbal Recognition Memory, Delayed Recall | Cambridge Neuropsychological Test Automated Battery | episodic |
| verbal fluency | verbal fluency test | semantic |
| Semantic memory | Boston Naming Task | semantic |
| verbal fluency | initial letter fluency | semantic |
| verbal fluency | excluded letter fluency | semantic |
| word fluency animals | verbal fluency test | semantic |
| word fluency letter p | verbal fluency test | semantic |
| word fluency letter s | Verbal fluency test | semantic |
| fluency names words | Verbal fluency test | semantic |
| corsi blocks span | corsi blocks task | working |
| spatial working memory | Cognitive Drug Research computerized assessment system | working |
| telephone number task | 9 digit phone number | working |
| three-back task | 45 letters 15 pairs | working |
| numeric working memory | 5 random digits | working |
| alphabetic working memory | 5 random letters | working |
| backward digit span | digit span backward | working |
| backward digit span | Wechsler Adult Intelligence Scale- revised | working |
| numeric working memory | Cognitive Drug Research computerized assessment system | working |
| Working Memory | letter number sequencing | working |
| Working Memory | digit span backward | working |
| Working Memory | letter number sequencing | working |
| Working Memory | digit span backward | working |
| n-back | Computerized mental performance assessment system | working |
| Spatial Working Memory | Cambridge Neuropsychological Test Automated Battery | working |
