# Supplementary material for: Docosahexaenoic Acid and Adult Memory: A Systematic Review and Meta-Analysis
Source: PLoS One. 2015 Mar 18;10(3):e0120391. doi: 10.1371/journal.pone.0120391 (PMC4364972; doi:10.1371/journal.pone.0120391)
Supplement: S2 Table — (DOCX) [file pone.0120391.s005.docx]

*S2 Table. Characteristics of Observational Studies Reporting DHA/EPA Status and Memory Outcomes*

| **Study** | **Country** | **Study Design** | **Baseline Cognitive Status^a^**  **(sample size)** | **Age**  **Range**  **(years)** | **DHA/EPA^b^ Status**  **(intake/issue – unit)** | **Follow-up** |
| --- | --- | --- | --- | --- | --- | --- |
| Beydoun, 2007 | U.S. | Prospective Cohort  (Atherosclerois Risk in Communities Study) | NCC  (2111)  MMC  (140) | 50-65 | NCC  0.55 / 0.45^¶^  0.57 / 2.87^§^  MMC  0.57 / 0.47*  0.57 / 2.98**  (^¶^pl CE %TFA^c^ ^§^pl PL %TFA^d^) | 6 yr |
| de Groot, 2007 | Netherlands | Prospective Cohort | NCC  (54) | 20-40 | 0.75 / 3.22  (pl PL%) | 3, 15, & 22 weeks |
| Dullemeijer, 2007 | Netherlands | Cross-sectional  and Prospective Cohort  (Folic Acid and Carotid Intima-Media Thickness Trial) | NCC  (807) | 50-70 | 0.92 / 0.60  (% pl CE) | 3 yr |
| Kalmijn, 2004 | Netherlands | Cross-sectional  (Doetinchem Cohort Study) | NCC  (1450)  MMC  (163) | 45-70 | NCC  167  MCC  145  (EPA+DHA Intake -mg) | 6 yr |
| Kesse-Guyot, 2011 | France | Prospective Cohort  (Supplementation with Antioxidant Vitamins and Minerals study) | 3,924 (All Subjects) | ~ 64 | 0.15 / 0.28  (Intake - g/day) | 13 yr |
| Milte,  2011 | Australia | Case-control | NCC  29  MMC  50 | ≥ 65 | NCC  1.26 / 4.53  MMC  0.94 / 4.55  (RBC^e^ PL %TFA) | NA^f^ |
| Muldoon, 2010 | U.S. | Cross-sectional  (University of Pittsburgh Adult  Health and Behavior Project) | NCC  280 | 30-54 | 0.49 / 1.52  (serum PL %) | NA |
| Phillips, 2012 | U.K. | Case-control | NCC  (61)  MMC  (55) | 55-91 | NCC  1.80 / 4.46  MMC  1.40 / 3.95  (pl PC^g^ %) | NA |
| Samieri, 2011 | France | Prospective Cohort  (Bordeaux sample of the  three-City (3C) study) | NCC  (1228) | ≥ 65 | 1.01 / 2.39  (pl %) | 7 y |
| Tan 2012 | U.S. | Cross-sectional  (Framingham Offspring Study) | NCC  (1575) | 67 ± 9 | 4.7 (DHA Median)  5.3 (DHA + EPA Median)  (RBC wt%^h^) | ~3 months |
| Titova, 2013 | Sweden | Prospective Cohort  (Prospective Investigation of  the Vasculature in Uppsala Seniors) | NCC  (252) | 70 | 0.026 to 0.667  (Intake range - g/d) | 5 yr |
| Whalley, 2004 | Scotland | Cross-sectional | NCC  (423) | ~64 | FO^i^  1.1 / 5.4  NFO^j^  0.8 / 4.6  (RBC %) | ~ 50 yr |
| Whalley, 2008 | Scotland | Prospective Cohort | NCC  (113) | ~66 | APOE ε4  0.89 / 5.0  Non-APOE ε4  0.98 / 5.0  (RBC %) | ~ 3 y |

^a^MMC = Mild Memory Complaints, NCC = No Cognitive Complaints; ^b^DHA = Docosahexaenoic acid; EPA = Eicosapentaenoic Acid; ^c^pl CE %TFA = plasma cholesterol ester, percent of total fatty acids; ^d^pl PL %TFA = plasma phospholipid, percent of total fatty acids;^e^RBC = red blood cell; ^f^ NA = Not Applicable;^g^ PC = Phosphatidylcholine;^h^wt% = weight percent; ^i^ FO = Fish oil;^j^NFO = Non-Fish Oil
