# Supplementary material for: Docosahexaenoic Acid and Adult Memory: A Systematic Review and Meta-Analysis
Source: PLoS One. 2015 Mar 18;10(3):e0120391. doi: 10.1371/journal.pone.0120391 (PMC4364972; doi:10.1371/journal.pone.0120391)
Supplement: S3 Table — (DOCX) [file pone.0120391.s006.docx]

| **S3 Table. Summary of DHA/EPA Supplementation and Semantic Memory Outcomes in Adults** | | | | | |  |  |  |  |
| --- | --- | --- | --- | --- | --- | --- | --- | --- | --- |
| **Between Group** | **# Data Points** | **WGMD** | **Z-score** | **p-Value Z-score** | **p-H** | **Hedge's g WGMD** | **Hedge's Z-score** | **p-Value Hedge's Z** | **p-H** |
| Overall | 15 | -0.224 | -0.686 | 0.493 | 0.484 | -0.044 | -0.785 | 0.433 | 0.522 |
| All NCC | 9 | -0.326 | -0.919 | 0.358 | 0.396 | -0.066 | -1.134 | 0.257 | 0.439 |
| All MMC* | 6 | 0.798 | 0.758 | 0.448 | 0.534 | 0.140 | 0.87 | 0.384 | 0.595 |
|  |  |  |  |  |  |  |  |  |  |
| Age > 45 years** | 13 | -0.058 | -0.170 | 0.865 | 0.546 | -0.008 | -0.128 | 0.898 | 0.572 |
|  |  |  |  |  |  |  |  |  |  |
| DHA+EPA Intake ≤1g | 5 | -0.838 | -1.758 | 0.079 | 0.522 | -0.150 | -1.878 | 0.06 | 0.605 |
| DHA+EPA Intake >1g | 10 | 0.316 | 0.708 | 0.479 | 0.616 | 0.055 | 0.727 | 0.467 | 0.648 |
|  |  |  |  |  |  |  |  |  |  |
| **Within Group** |  |  |  |  |  |  |  |  |  |
| All studies | 15 | 0.583 | 2.400 | **0.016** | 0.376 | 0.092 | 2.357 | **0.018** | 0.478 |
| All NCC | 9 | 0.552 | 1.887 | 0.059 | 0.22 | 0.085 | 1.824 | 0.068 | 0.301 |
| All MMC* | 6 | 1.183 | 1.787 | 0.074 | 0.656 | 0.177 | 1.684 | 0.092 | 0.646 |
|  |  |  |  |  |  |  |  |  |  |
| Age >45 years** | 13 | 0.586 | 2.107 | **0.035** | 0.278 | 0.09 | 1.971 | **0.049** | 0.35 |
|  |  |  |  |  |  |  |  |  |  |
| DHA+EPA Intake ≤1g | 5 | 0.635 | 1.158 | 0.247 | 0.061 | 0.085 | 0.996 | 0.319 | 0.097 |
| DHA+EPA Intake >1g | 10 | 0.79 | 2.504 | **0.012** | 0.859 | 0.13 | 2.435 | **0.015** | 0.862 |
|  |  |  |  |  |  |  |  |  |  |
| *Semantic memory outcomes for subjects with MMC reported only by Sinn et al.; ** Includes all studies but Jackson, the only study reporting semantic memory outcomes in subjects ≤ 45 years of age; NCC = subjects without cognitive complaints at baseline; MMC = subjects with mild memory complaints at baseline | | | | | | | |  |  |

WGMD = weighted group mean difference; p-H = p-value for heterogeneity test; NCC = subjects without cognitive complaints at baseline; MMC = subjects with mild memory complaints at baseline
