# Supplementary material for: Docosahexaenoic Acid and Adult Memory: A Systematic Review and Meta-Analysis
Source: PLoS One. 2015 Mar 18;10(3):e0120391. doi: 10.1371/journal.pone.0120391 (PMC4364972; doi:10.1371/journal.pone.0120391)
Supplement: S4 Table — (DOCX) [file pone.0120391.s007.docx]

| **S4 Table. Summary of DHA/EPA supplementation and working memory outcomes in adults** | | | | | | |  |  |  |
| --- | --- | --- | --- | --- | --- | --- | --- | --- | --- |
| **Between Group** | **# Data Points** | **WGMD** | **Z-score** | **p-Value Z-score** | **p-H** | **Hedge's g WGMD** | **Hedge's Z-score** | **p-Value Hedge's Z** | **p-H** |
| Overall | 21 | 0.023 | 0.167 | 0.867 | 0.112 | 0.031 | 0.601 | 0.548 | 0.161 |
| All NCC | 15 | -0.042 | -0.380 | 0.704 | 0.471 | 0.010 | 0.19 | 0.849 | 0.472 |
| All MMC | 6 | 0.234 | 0.417 | 0.677 | 0.017 | 0.051 | 0.267 | 0.79 | 0.043 |
|  |  |  |  |  |  |  |  |  |  |
| Age ≤ 45 years | 11 | 0.053 | 0.341 | 0.733 | 0.345 | 0.053 | 0.866 | 0.387 | 0.407 |
| Age >45 years | 10 | 0.037 | 0.146 | 0.884 | 0.064 | 0.008 | 0.081 | 0.936 | 0.076 |
|  |  |  |  |  |  |  |  |  |  |
| DHA+EPA Intakes ≤1g | 14 | -0.01 | -0.080 | 0.936 | 0.495 | 0.042 | 0.859 | 0.39 | 0.564 |
| DHA+EPA Intakes >1g | 7 | 0.066 | 0.182 | 0.856 | 0.017 | 0.020 | 0.126 | 0.9 | 0.024 |
|  |  |  |  |  |  |  |  |  |  |
| **Within Group** |  |  |  |  |  |  |  |  |  |
| Overall | 23 | 0.183 | 2.396 | **0.017** | 0.565 | 0.066 | 2.21 | **0.027** | 0.642 |
| All NCC | 15 | 0.146 | 1.798 | 0.072 | 0.626 | 0.069 | 1.922 | 0.055 | 0.693 |
| All MMC | 8 | 0.463 | 2.07 | 0.038 | 0.455 | 0.087 | 1.162 | 0.245 | 0.322 |
|  |  |  |  |  |  |  |  |  |  |
| Age ≤45 years | 11 | 0.061 | 0.578 | 0.563 | 0.451 | 0.039 | 0.93 | 0.352 | 0.529 |
| Age >45 years | 12 | 0.314 | 2.852 | **0.004** | 0.742 | 0.092 | 2.193 | **0.028** | 0.6 |
|  |  |  |  |  |  |  |  |  |  |
| DHA+EPA Intake ≤1g | 16 | 0.067 | 0.716 | 0.474 | 0.966 | 0.024 | 0.702 | 0.482 | 0.968 |
| DHA+EPA Intake >1g | 7 | 0.503 | 2.655 | **0.008** | 0.18 | 0.208 | 3.34 | **0.001** | 0.459 |
|  |  |  |  |  |  |  |  |  |  |
|  |  |  |  |  |  |  |  |  |  |
